# Ultrahigh-resolution, high-fidelity quantum dot pixels patterned by dielectric electrophoretic deposition

**DOI:** 10.1038/s41377-024-01601-3

**Published:** 2024-09-26

**Authors:** Chengzhao Luo, Yanhui Ding, Zhenwei Ren, Chenglong Wu, Yonghuan Huo, Xin Zhou, Zhiyong Zheng, Xinwen Wang, Yu Chen

**Affiliations:** 1 School of Optoelectronic Science and Engineering & Collaborative Innovation Center of Suzhou Nano Science and Technology, Soochow University, Suzhou, 215006 China; 2 National University of Singapore Suzhou Research Institute, Dushu Lake Science and Education Innovation District, Suzhou, 215123 China

**Keywords:** Lasers, LEDs and light sources, Optical materials and structures

## Abstract

The high pixel resolution is emerging as one of the key parameters for the next-generation displays. Despite the development of various quantum dot (QD) patterning techniques, achieving ultrahigh-resolution (>10,000 pixels per inch (PPI)) and high-fidelity QD patterns is still a tough challenge that needs to be addressed urgently. Here, we propose a novel and effective approach of orthogonal electric field-induced template-assisted dielectric electrophoretic deposition to successfully achieve one of the highest pixel resolutions of 23090 (PPI) with a high fidelity of up to 99%. Meanwhile, the proposed strategy is compatible with the preparation of QD pixels based on perovskite CsPbBr_3_ and conventional CdSe QDs, exhibiting a wide applicability for QD pixel fabrication. Notably, we further demonstrate the great value of our approach to achieve efficiently electroluminescent QD pixels with a peak external quantum efficiency of 16.5%. Consequently, this work provides a general approach for realizing ultrahigh-resolution and high-fidelity patterns based on various QDs and a novel method for fabricating QD-patterned devices with high performance.

## Abstract (web summary)

The orthogonal electric field-induced template-assisted dielectric electrophoretic deposition is proposed to achieve high pixel resolution (23090 PPI) with a high fidelity (99%), which is further demonstrated for efficient electroluminescent devices.

## Graphical abstract

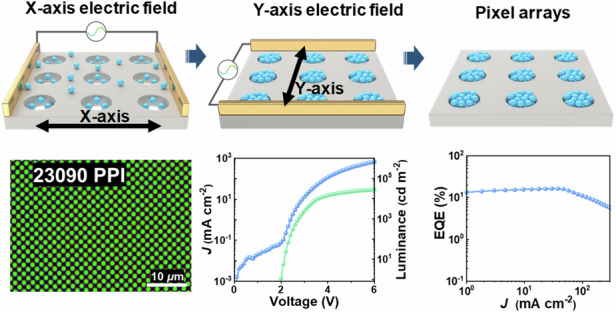

## Introduction

Colloidal quantum dots (QDs) have emerged as one of the most competitive emitters for applications in high-performance displays due to their excellent optical properties, such as tunable emission wavelengths, narrow emission spectra, and high luminescent efficiency^1–6^. Nowadays, ultrahigh-resolution QD pixel patterns with a pixel resolution beyond 10,000 pixels per inch (PPI) are eagerly desired to meet the requirements of next-generation displays, such as virtual reality, three-dimensional (3D) displays, and near-eye displays, which are featured with high information flux by redistributing graphical information from 2D screen into 3D space^7^. Unfortunately, there are limited studies involving the fabrication of ultrahigh-resolution QD pixel patterns with prominent electroluminescence (EL) properties. More importantly, the QD pixel fidelity deteriorates with improving the QD pixel resolution, thus it remains a big challenge to achieve ultrahigh-resolution QD pixel patterns with high fidelity, as well as the efficient quantum dot light-emitting diodes (QLEDs)^8,9^.

Being different from the patterned organic material pixels with a thermal-evaporated fabrication process^10^, the QD pixels are mainly achieved through solution-compatible approaches, such as inkjet printing, transfer printing, and photolithography^11–13^. In particular, the inkjet printing method has been extensively employed to fabricate patterned QD pixels due to its good compatibility with QD inks. While some issues are encountered during the preparation of QD pixels with the inkjet printing method. Specifically, the QD pixel resolution is greatly restricted by the size of the inkjet print head, resulting in limited resolution of several hundreds of PPI with pixels of dozens of micrometers^14,15^. In addition, a common phenomenon of coffee rings during the QD fabrication process with the inkjet printing process causes non-uniform QD pixels for poor emission efficiency^16^. Compared with the inkjet printing method, the technique of transfer printing provides an effective way to improve the QD pixel resolution, thus being also widely adopted to prepare high-resolution QD patterns^17,18^. Very recently, micro-honeycomb QD patterns with an impressive pixel resolution of 25400 PPI have been reported by combining the transfer printing technique with the Langmuir–Blodgett film technology^19^. However, there is a concern about the exact alignment of the micron-sized patterns during the transfer printing process. In other words, the issue of difficult alignment of the micron-sized patterns would result in poor fidelity of the high-resolution QD patterns. Meanwhile, the sophisticated templates used in transfer printing may be much complex and costly. Those problems pose some difficulties in the preparation of high-resolution and high-fidelity QD patterns with the transfer printing technique.

Besides, the method of photolithography is also widely employed to fabricate QD patterns with high fidelity and various shapes/dimensions. While in conventional photolithography, there is an inevitable step to cover the QDs with the photoresist during the patterned film fabrication process^20^. Unfortunately, both the photoresist solution and the following process with the photographic developer induce permanent damage to the underlying QDs layer, thus resulting in poor luminescent efficiency^21^. In other words, the issue of QD damage creates substantial difficulties in the fabrication of high-quality QD patterns with conventional photolithography. To solve this problem, the method of post-deposition of QDs on the patterned substrates provides an effective way to obtain high-quality QD patterns^22^. For example, the electrostatic force-induced deposition was developed in our previous work to obtain highly emissive and accurate QD patterns by introducing alternating polyethyleneimine and fluorosilane patterns on the substrates prepared via lithographic process^23^. Alternatively, the selective electrophoretic deposition (SEPD) combined photolithography was also demonstrated to precisely deposit the QDs on the determined polarity electrodes for large-area QD arrays^24^. The surface morphology, packing density, and refractive index of the QD patterns can be well-tuned by the electric field during an electrophoretic deposition process, showing a decent way to tailor the corresponding QD device performance. While the QD patterns obtained from SEPD are greatly restricted by the photolithographic electrodes. In other words, the QD arrays are typically continuous patterns, which may limit the application of SEPD in practical use. Therefore, it is necessary to develop a new approach for effectively manipulating the QDs for high-resolution patterns.

In this work, we propose a novel and effective approach of orthogonal electric field-induced template-assisted dielectric electrophoretic deposition (DED) for high-resolution and high-fidelity QD pixel fabrication. Based on the templates with submicron notches, an ultrahigh-resolution of 23,090 PPI, being one of the highest reported resolutions for QD pixels, and a high fidelity of 99% are successfully achieved. The proposed strategy can be adapted for the QD pixels with perovskite CsPbBr_3_ QDs and the conventional CdSe QDs, showing its wide applicability for QD pixel fabrication. Furthermore, we the demonstrate the versatility of our approach to achieve highly electroluminescent QD pixels with a high luminance of 27,861 cd m^−2^ and a peak external quantum efficiency (EQE) of 16.5% through the suppression of the leakage current between pixels with poly-dimethylsiloxane (PDMS), showing great potential for our strategy in the preparation of high-performance QD-patterned devices toward practical application.

## Results

### QD optoelectronic properties

**Fig. 1:**
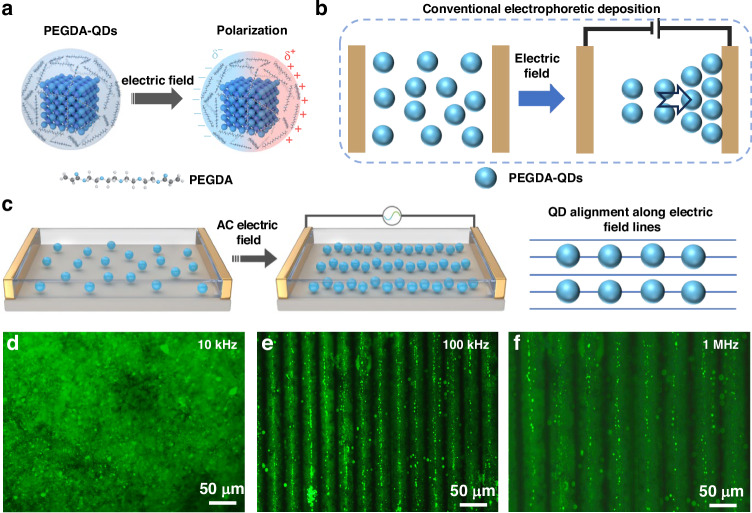
Dielectric electrophoretic deposition process. Schematic diagrams of **a** the polarization of PEGDA-QDs under an electric field, **b** the conventional electrophoretic deposition, and **c** the dielectric electrophoretic deposition (DED) process of QDs. Fluorescence microscopy images of QD patterns under various electric field frequencies of **d** 10 kHz, **e** 100 kHz, and **f** 1 MHz, respectively

To start with the DED, the CsPbBr_3_ QDs with green emission were first synthesized through the facile synthetic process. While the QD surface is typically surrounded by dense and long-chain organic ligands, such as oleic acid, oleylamine, didodecyl dimethylammonium, etc., which induce a low dielectric constant for the QDs, thus low polarizability under an electric field (Supplementary Fig. S1). Meanwhile, the perovskite QDs were reported to show poor stability against the volatile electric field, which undoubtedly sets an obstacle to their application in dielectric electrophoresis^25^. To improve the QD polarizability and stability under an electric field, the polymer of poly(ethylene glycol) diacrylate (PEGDA) was employed to decorate the QDs during the synthesis process. Details are shown in the Supplementary Information. The PEGDA-decorated QDs (PEGDA-QDs) have a high photoluminescence quantum yield (PLQY) of 83.2%, enabling a bright green emission (Supplementary Fig. S2). Meanwhile, the capping role of PEGDA for the QDs was also investigated. As shown in Fourier transform infrared spectroscopy (FT-IR) (Supplementary Fig. S3), the characteristic peak centered at 3465 cm^−1^ for the QDs can be assigned to the stretching vibration of N–H from the alkylamine ligand. While the peak disappears after the PEGDA decoration, and instead, the PEGDA signals (e.g., the stretching vibration peak of 1724 cm^−1^ attributed to the acrylate from PEGDA) are clearly presented in PEGDA-QDs, showing a good capping effect of PEGDA. Furthermore, the PEGDA-QDs exhibit a larger dielectric constant than that of pure QDs across the whole frequency range (Supplementary Fig. S1). The enlarged dielectric constant for PEGDA-QDs suggests that the PEGDA-QDs can be facilely polarized under an electric field (Fig. 1a). Then the prepared PEGDA-QDs were dispersed in a mixed solution consisting of *n*-hexane and dimethylsiloxane (DMS) oligomer for further use. Here, the DMS oligomer was incorporated due to its facile polymerization under a mild heating process, which benefits the inhibition of the current leakage, as discussed in the following part.

### Dielectric electrophoretic deposition process analysis

In conventional electrophoretic deposition, the QDs are randomly distributed in the solution without an electric field. While the QDs are driven to migrate and aggregate on the electrodes by the applied direct current (DC) electric field (Fig. 1b)^25–27^. Differently, in DED, the QDs are generally driven by alternating electric field to move in a translational motion along the electric field gradient and align in the direction of the electric field lines, as illustrated in Fig. 1c. Notably, the pristine charged QDs (Supplementary Table S1) cannot be directly driven by an alternating electric field for orientated motion due to their low polarizability under an electric field as shown in Supplementary Fig. S4. A satisfactory QD pattern can be fabricated by PEGDA-QDs with a large dielectric constant during DED process, highlighting the distinction between this proposed DED and the conventional electrophoretic deposition. Interestingly, it is found that the QD-aligned patterns are closely associated with the frequency of the applied voltage. When the frequency was set at 10 kHz, bright luminescence was observed throughout the viewing area in the fluorescence microscopy image (Fig. 1d), and no QD alignment was formed. While increasing the frequency to 100 kHz, distinct aligned patterns with strong green emission were observed, showing that the QDs were driven along the electric field gradient and self-assembled into linear patterns (Fig. 1e). Besides, the dark area between the adjacent aligned patterns induced by the QD-orientated motion was also clearly observed. When the voltage frequency further increased to 1 MHz, the QD-aligned linear patterns were widened to 47.1 μm, accompanied by a weakened luminescence (Fig. 1f).

The variation of QD alignment width with electric field frequency can be clarified by the dielectric electrophoretic force (*F*_DE_) which drives the QD translational motion under an electric field. The value of *F*_DEP_ can be determined by the equation of *F*_DE_ = 2π*R*^3^*ε*_m_Re[*f*(*ω*)] ∇*E*^2^, where *R* is the radius of QDs, *ε*_m_ is the solvent permittivity, *ω* is the angular frequency, *E* is the electric field intensity, and Re[*f*(*ω*)] is the real part of the Clausius–Mossotti factor, respectively^25,28^. According to the equation, the *F*_DE_ is proportional to the value of Re[*f*(*ω*)] at a constant electric field intensity. While the Re[*f*(*ω*)] value is related to the electric field frequency. In detail, the Clausius–Mossotti factor can be derived from the equation of *f*(*ω*) = (*ε*_p_^*^(*ω*) − *ε*_m_^*^(*ω*))/(*ε*_p_^*^(*ω*) + 2*ε*_m_^*^(*ω*)), where *ε*_p_^*^(*ω*) and *ε*_m_^*^(*ω*) are the complex permittivities of the QDs and the solvent, respectively. The complex permittivity can be calculated from the equation of ε^*^ = *ε* − *i*(σ/*ω*), in which *i* is the imaginary number, *ε* is the permittivity of the QDs and the solution, and *σ* is the conductivity of the QDs and the solution, respectively^26,29^. The relationship between Re[*f*(*ω*)] and the electric field frequency is plotted in Supplementary Fig. S5, where the Re[*f*(*ω*)] value increases with improving the electric field frequency and reaches the maximum value at 146 kHz. Then the Re[*f*(*ω*)] declines with further increasing the voltage frequency. Due to the dependent relationship between *F*_DE_ and Re[*f*(*ω*)], the *F*_DE_ value will increase with increasing the frequency from 10 to 146 kHz and decrease at higher frequencies. The variation in *F*_DE_ well explains the changes in QD-aligned patterns at different frequencies. Additionally, the *F*_DE_ is also proportional to the electric field intensity based on the equation of *F*_DEP_. To further verify the oriented QD movement manipulated by *F*_DE_, the effect of electric field intensity on QD motion was investigated. As shown in Supplementary Fig. S6, when increasing the electric field intensity from 4 to 8 V mm^−1^, the QDs converged with bright emission along the electric field direction, accompanied by the widened dark area between the adjacent alignment patterns. Besides, the evolutions of QD patterns at different DED times were also monitored (Supplementary Fig. S7). It is found that the QDs gradually self-assembled into linear patterns with increasing the deposition time from 30 s to 10 min. In particular, the distinct aligned patterns with strong green emission were achieved at the deposition time of 10 min, accompanied by a dark area between the adjacent aligned patterns, showing the effective QD-orientated motion driven by the dielectric electrophoretic force within 10 min. The above results vividly show the effective manipulation of QD-aligned patterns by *F*_DE_.

### Regular and precise QD pattern fabrication

**Fig. 2:**
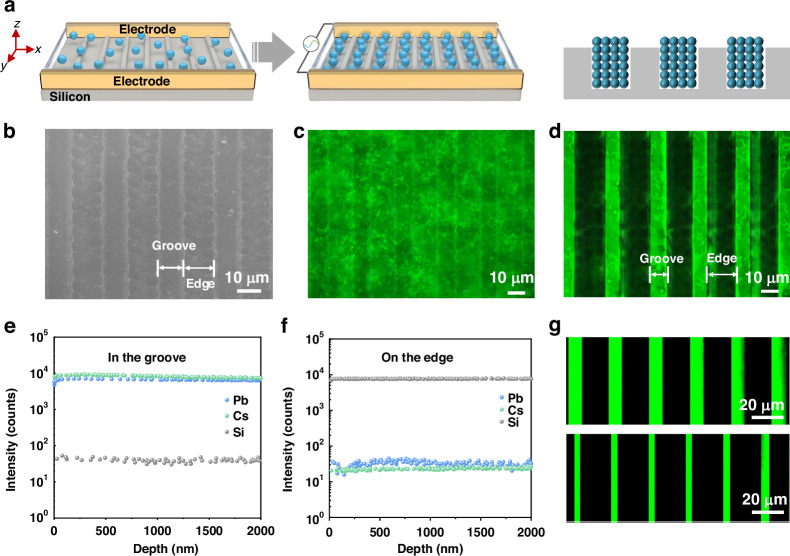
Template-assisted DED process. **a** Schematic diagram of the stripe template-assisted DED process. **b** Scanning electron microscopy (SEM) image of the stripe templates and fluorescence microscopy images of QD patterns on the templates **c** without and **d** with the electric field (4 V mm^−1^, 100 kHz). Dynamic secondary ion mass spectrometry (SIMS) of the elements of Pb, Cs, and Si in the areas of the **e** groove and **f** edge on the templates. **g** Fluorescence microscopy images of QD patterns with the QDs concentrations at 20 (top) and 15 (down) mg mL^−1^, respectively

The *F*_DE_-manipulated QD patterns are considerably loose in assembling the QD pixels for the application in displays. In order to obtain regular and precise QD patterns, the template-assisted DED was developed. As illustrated in Fig. 2a, the templates with stripe grooves were employed as the substrates for QD deposition. When an alternating electric field was applied to the templates, the QDs were driven to move translationally and accumulate in the grooves. As verified in Fig. 2b, when the QD solution was dropped on the substrates with grooves of around 10 μm in width, there was a bright emission over the whole area of the substrates (Fig. 2c), showing a good coverage of the QDs on the substrates. The looming patterns of the template in Fig. 2c after covering the QD solution can be attributed to the projection of the UV light. When the bias voltage (4 V mm^−1^, 100 kHz) was applied, the regular QD stripes with the same width as that of the grooves were achieved (Fig. 2d). To validate the migration of QDs to the grooves, dynamic secondary ion mass spectrometry (SIMS) was performed on the QD-aligned templates. In the groove area, the strong Cs and Pb signals from CsPbBr_3_ QDs were detected, meanwhile, the Si signal merely showed a weak intensity (Fig. 2e), indicating the accumulation of the QDs in the grooves. In addition, the Cs and Pb signals showed nearly unchanged intensity with increasing the detected depth to 2 μm, showing a homogeneous distribution of the QDs in the grooves. In contrast to the observation in grooves, the Cs and Pb signals on the template edge exhibited weak signals with the detected depth increased from 0 to 2 μm (Fig. 2f), instead, a strong intensity was found for the Si signal, further verifying the migration of QDs to the grooves. Notably, the width of the QD-aligned patterns can be tuned by changing the QD concentrations. As shown in Fig. 2g, when the QDs concentration decreases from 20 to 15 mg mL^−1^, the width is narrowed from 9 to 4.5 μm due to the reduced quantity of QDs. To further verify the proposed template-assisted DED for QD-aligned patterns, an array of different QD stripe patterns was fabricated by altering the template geometric shapes (Supplementary Fig. S8). Consequently, we have shown the template-assisted DED for stripe QD patterns with regular and precise QD patterns, which paves an effective approach to manipulate the QD deposition.

### Ultrahigh-resolution and high-fidelity QD pixel fabrication

**Fig. 3:**
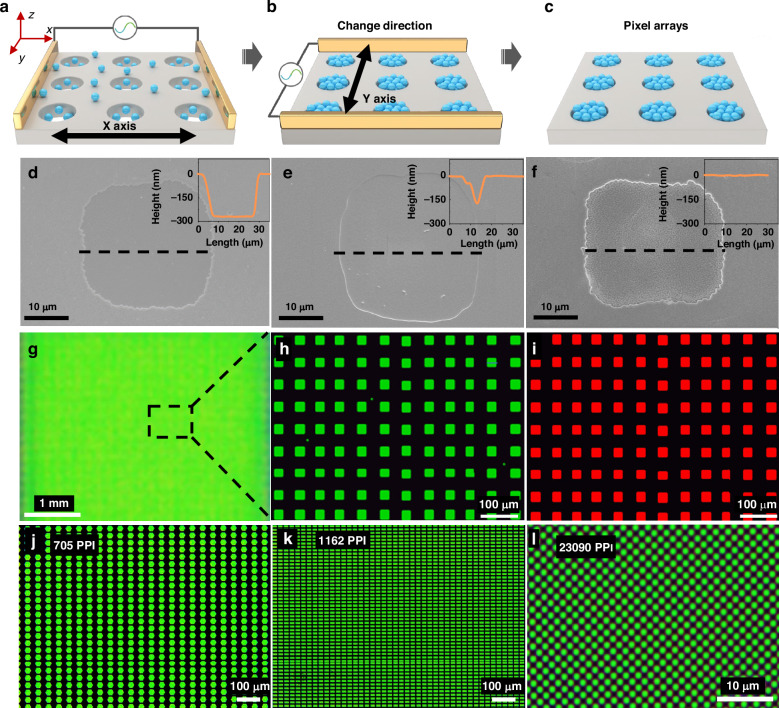
QD pixel fabrication. Schematic diagram of the template-assisted QD pixel deposition process successively driven by **a** *X*- and **b** *Y*-axis electric field, **c** QD pixel patterns after the orthogonal electric field, and **d**–**f** the corresponding scanning electron microscopy (SEM) images of the square grooves after applying the electric field (inset: the groove depth along the dotted line characterized by a step profiler). Fluorescence microscopy images of **g** the CsPbBr_3_ QD pixels and the **h** enlarged view of the rectangular area in (**g**), **i** red CdSe QD pixels. **j**–**l** Different QD pixel resolutions fabricated by the proposed strategy with around 23, 14, and 0.8 μm in length for the QD resolutions of 705, 1162, and 23,090 PPI, respectively

Furthermore, inspired by the striped QD patterns fabricated by our developed template-assisted DED method, the fabrication of QD pixels was successively investigated. The templates with an array of square (circular) shapes were adopted as the substrates for the QD pixel fabrication (Supplementary Fig. S9). Similar to the process for stripe QD patterns, the QD solution was first dropped on the substrates. Then the alternating electric field along the *X*-axis was applied, where the QDs were driven to align along the *X*-axis with partial accumulation of QDs in the circular grooves (Fig. 3a). The oriented QD motion toward the grooves is confirmed by the variation of the groove depth. As shown in Fig. 3d, the initial groove depth characterized by a step profiler was 270 nm, which sharply decreased with the maximum depth declining to 173 nm after exposure to an alternating electric field along the *X*-axis for 5 min (Fig. 3e), indicating the QD accumulation in the grooves. Afterward, the alternating electric field in *Y*-axis was applied to drive the QD alignment along the *Y*-axis (Fig. 3b), resulting in a continuous QD accumulation into the grooves as evidenced by the progressively decreased groove depth to zero (Fig. 3f). As a result, the 0 D grooves were filled with the QDs driven by the orthogonal electric field (Fig. 3c). To further confirm the effective alignment and accumulation of QDs in the grooves, the PL intensities of the QD pixels and the void spaces in-between were detected. As shown in Supplementary Fig. S10, the areas between pixels show no emission within the detection view, whereas the QD pixels exhibit bright emissions, suggesting our proposed DED an effective QDs manipulation approach for pixel construction. Based on the template-assisted DED with the orthogonal electric field, the large-area QD pixels were successfully fabricated (Fig. 3g). The magnified fluorescence microscopy image in Fig. 3h shows the square-shaped pixels with a size of 25 µm. To further verify our developed template-assisted DED for QD pixel fabrication, the pixels with different geometric shapes (e.g., rectangle and regular hexagon shapes) were also successfully achieved (Supplementary Fig. S11). Meanwhile, we also achieved the red-emissive pixels-based CdSe QDs based on our proposed strategy (Fig. 3i), demonstrating good compatibility for QD pixel fabrication with different types of QDs.

Since the QD pixel resolution is a crucial factor for QD patterns, the preparation of high-resolution QD pixels was also investigated. As shown in Fig. 3j, the regular hexagon and rectangle-shaped QD patterns with resolutions of 705 and 1162 PPI can be handily fabricated based on the proposed template-assisted DED method. To further verify the capability of our method for ultrahigh-resolution QD pixels, such as submicron patterns, the polystyrene (PS) sphere-masked technique in combination with the inductively coupled plasma (ICP) etching process was employed to prepare submicron notches (Supplementary Fig. S12)^30^. Details are shown in the Supplementary Information. Eventually, based on the elaborated template, submicron QD pixels with a diameter of around 0.8 μm were achieved, enabling an ultrahigh-resolution of 23,090 PPI (Fig. 3l), which represents one of the highest reported resolutions for QD pixels (Table 1).

**Table 1:** High-resolution CdSe QD pixel comparisons

Pattern technology	Resolution	Pixel completeness	QD pixel fidelity	EQE	Published journals
Photolithography	1400 PPI	Ideal: circle Actual:	Unsatisfied	14.6%	*Nat. Commun*. 11, 2874 (2020)
Photolithography	1500 PPI	Ideal: square Actual:	Satisfied	19.1%	*Nano Lett*. 23, 2000 (2023)
Programmed microwetting	1953 PPI	Ideal: rectange Actual:	Unsatisfied	/	*ACS Nano* 16, 16598 (2022)
Intaglio transfer printing	2460 PPI	Ideal: square Actual:	Satisfied	2.35%	*Nat. Commun*. 6, 7149 (2015)
Electrostatic force-induced deposition	3031 PPI	Ideal: square Actual:	Satisfied	15.6%	*Adv. Mater*. 35, 2303329 (2023)
Photolithography	15,000 PPI	Ideal: square Actual:	Satisfied	/	*Nat. Nanotechnol*. 17, 952 (2022)
Transfer printing	25,400 PPI	Ideal: circle Actual:	Unsatisfied	9072 PPI, 14.72%	*Nat. Photonics* 16, 297 (2022)
Thermodynamic-driven	14,063 PPI	Ideal: rectange Actual:	Unsatisfied	3.3%	*Nat. Commun*. 11, 3040 (2020)
Orthogonal alternating electric field	23,090 PPI	Ideal: circle Actual:	99%	254 PPI, 16.5% 507 PPI, 14.1% 1037 PPI, 11.7%	This work

Furthermore, the QD pixel fidelity is also an important parameter to evaluate the QD pattern quality which is defined as the ratio of the QD pixel area to the mask area on the template (Supplementary Fig. S13). As shown in the previous report, the QD pixel fidelity sharply declines with an appearance of irregular shapes at the pixel resolutions beyond thousands^19^. Encouragingly, a high fidelity of 99% was achieved for our ultrahigh-resolution QD pixels (23,090 PPI), showing excellent exactness to the original patterns. In all, we have proven the ultrahigh-resolution and high-fidelity QD pixels achieved with the proposed strategy of orthogonal electric field-induced template-assisted DED, which contributes a new and effective approach to fabricating the elaborated QD pixels toward practical application.

### Device performance

**Fig. 4:**
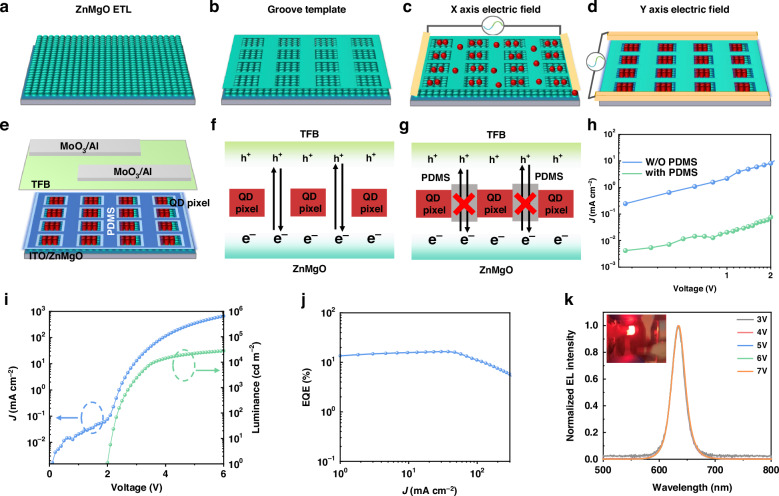
Device performance. **a**–**d** Schematic of the fabrication process of QD pixel-based QLEDs, and **e** the device structure. Illustration of the leakage current without (**f**) and with (**g**) PDMS. **h** The current density–voltage (*J*–*V*) curves of the devices with and without PDMS. The device performances: **i** current density–luminance–voltage (*J*–*L*–*V*) and **j** efficiency–current density (*EQE*–*J*) curves. **k** The normalized EL spectra under different voltages (inset: the picture of the device during the test)

Although substantial progress has been made to obtain various QD pixels with the main focus on their photoluminescence (PL) performance^31–34^, the electroluminescent performance of the QD pixels is challenging to evaluate due to the incompatible fabrication process for the devices. Notably, we demonstrate the versatility of our approach to achieve efficiently electroluminescent QD pixels. In detail, the magnesium-doped zinc oxide (ZnMgO) nanoparticles, serving as the electron transport layer (ETL), were first deposited on the indium tin oxide (ITO) cathode (Fig. 4a). Subsequently, the grooves were prepared on ZnMgO layer by photolithography to serve as the templates for the following QD deposition (Fig. 4b). Afterward, the emissive layer of CdSe QD pixels was prepared by applying an orthogonal electric field (Fig. 4c, d). Then the layers of poly(9,9-dioctylfluorene-co-*N*-(4-butylphenyl)-diphenylamine) (TFB) and MoO_3_/Al were successively deposited to complete the device configuration of ITO/ZnMgO/QD patterns/TFB/MoO_3_/Al (Fig. 4e). As extensively reported in previous studies^19,22,35,36^, the issue of leakage current in QD pixel-based devices, caused by the void spaces among QD pixels, significantly limits the device performance (Fig. 4f). However, the leakage current can be effectively suppressed by introducing DMS oligomer into the QD solution. As illustrated in Fig. 4g, the oligomer can polymerize into PDMS under a mild heating process (e.g., 70 °C, 10 min), exhibiting sufficient stability and good wettability to the chlorobenzene solvent (Supplementary Figs. S14 and S15), serving as a suitable substrate for subsequent device fabrication. For a comparison, the devices without PDMS were fabricated, which show high current densities versus the applied voltages due to the serious current leakage as further verified by the strong blue emission from TFB (Supplementary Fig. 16c), resulting in a poor device efficiency (Supplementary Fig. S16b). Conversely, the PDMS-based devices have lower current densities at the same voltage, confirming the effective inhibition of the leakage current (Fig. 4h). The reduced leakage current enables satisfactory device performances with a high luminance of 27861 cd m^−2^ (Fig. 4i) and a peak EQE of 16.5% (Fig. 4j) at a pixel resolution of 254 PPI, representing one of the highest efficiencies for pixelated QLEDs^10,20,36–40^. In addition, the QLED performances based on different QD pixel resolutions were investigated (Supplementary Fig. S17), where the device with 254 PPI shows the highest efficiency. The device performances decrease to 14.1 and 11.7% with increasing QD resolutions to 507 and 1037 PPI, respectively, being ascribed to the deteriorated charge transport property at higher resolutions. Meanwhile, the devices exhibit a clear EL emission for the individual pixels with no emission detected in the void spaces (Supplementary Fig. S18). The observation is consistent with the results of PL intensity measurements, validating our proposed DED an effective method for pixel construction. The QLEDs also show good spectral stability, in which the peak position of 634 nm and full width at half maximum (FWHM) of 27 nm remain unchanged at various applied voltages up to 7 V (Fig. 4k). In addition, the EL peak of 634 nm is much approaching the QD PL peak position of 632 nm, indicating that the QD optical property remains consistent after the QLED fabrication process. The slight red shift of 2 nm for the EL peak can be ascribed to the quantum-limited Stark effect, as reported previously^41–43^. Consequently, the strategy of template-assisted DED has been demonstrated as an effective approach for high-performance QLEDs by addressing the issue of leakage current, and a peak EQE of 16.5%, one of the most efficient pixelated QLEDs has been achieved, showing a great potential application in the preparation of efficient QD-patterned devices toward practical use.

## Discussion

We have developed a new and effective strategy of orthogonal electric field-induced template-assisted DED for the fabrication of QD patterns. Based on our homemade submicron notches template, an ultrahigh-resolution of 23,090 PPI, one of the highest reported resolutions for QD pixels has been achieved to address the challenge in ultrahigh-resolution QD pixel fabrication with conventional approaches, such as photolithography, intaglio transfer printing, electrophoretic deposition, etc. More importantly, we achieve a high fidelity of 99% for the ultrahigh-resolution QD pixels, solving the tough issue of poor fidelity in high-resolution QD pixels with conventional methods. Meanwhile, our proposed strategy has also shown its good compatibility for QD pixel fabrication with wide applicability for both perovskite and CdSe QDs. In addition, we demonstrate the great value of our approach to prepare QD-patterned devices with high electroluminescent performance. The constructed QLEDs exhibit impressive device performances with peak EQEs of 16.5, 14.1, and 11.7% for 254 PPI, 507 PPI, and 1037 PPI pixelated QLEDs by suppression of the leakage current with PDMS between micro-sized pixels. These results confirm our strategy to be an effective approach to fabricate ultrahigh-resolution and high-fidelity QD pixels, as well as high-performance QD-patterned devices for great potential application in commercialized displays.

## Materials and methods

### Materials

The DMS used in the experiment was Sylgard 184 purchased from Aladdin. PEGDA with a relative molecular mass of 600, lead bromide (PbBr_2_, 98%), didodecyl dimethylammonium bromide (DDAB, 98%), and methyl acetate (98%) were purchased from Aladdin Co., Ltd. The reagents used were hexane (99.9%) and toluene (99.9%), both purchased from Macklin Co., Ltd. Tetraoctylammonium bromide (TOAB, 98%), cesium carbonate (Cs_2_CO_3_, 98%), and octanoic acid (OTAc, 99.9%) were purchased from Macklin Co., Ltd. The monocrystalline silicon wafers (both the length and width are 2.5 cm, the thickness is 0.75 cm) used for etching were purchased from Luoyang Jingchen Electronic Technology Co., Ltd. The CdSe QDs were purchased from Suzhou Xingshuo Nanotechnology Co., Ltd. Monodispersed colloidal PS spheres in an aqueous solution (5 wt%) with different nominal diameters (i.e., 1 and 2 μm) were purchased from Shanghai Huge Biotechnology Co., Ltd. Poly[(9,9-dioctylfluorenyl-2,7-diyl)-co-(4,40-(N-(4-s-butylphenyl)) diphenylamine)] (TFB) was purchased from American Dye Source.

### Synthesis of PEGDA-decorated CsPbBr_3_ QDs

The PEGDA-decorated CsPbBr_3_ QDs were prepared at room temperature. Typically, 1 mL Cs_2_CO_3_ solution (0.1 mmol mL^−1^ in OTAc) was quickly added into 9 mL PbBr_2_ solution (0.1 mmol mL^−1^ in toluene, mixed with TOAB at a molar ratio of 1:2). The solution was stirred for 5 min at room temperature in air. Then 3 mL DDAB solution (10 mg mL^−1^ in toluene) and a proper amount of PEGDA (13 wt% to DDAB in toluene) were added and stirred for another 2 min. The PEGDA-CsPbBr_3_ QDs were obtained after the purification process with ethyl acetate. Then the PEGDA-CsPbBr_3_ QDs were dispersed in *n*-hexane for further use. For the PEGDA-decorated CdSe QDs, a proper amount of PEGDA (1.2 wt% to QDs) was added into the QDs solution (the solvent is toluene). After stirring for 15 min, the PEGDA-CdSe QDs were collected after the purification process with ethanol and toluene and then re-dispersed in *n*-hexane for further use.

### Preparation of the templates with microgrooves

The silicon substrates were successively cleaned with deionized water, acetone, and isopropyl alcohol. Then cleaned substrates were further treated with UV-ozone for 20 min to improve surface hydrophilicity. The photoresist (AZ5214) was spin-coated on the substrates and annealed at 100 °C in the air for 45 s. After cooling, the substrates were exposed to a 365 nm UV light for 18 s, followed by the developer process with a developer solution (AZ400k: H_2_O = 1:4, v/v) to remove the photoresist from the unexposed areas. Then the substrates were placed into an ICP etching equipment chamber containing 40 sccm of CF_4_ and 20 sccm of SF_6_ gases to etch the silicon for notched patterns. After that, the substrates were immersed in acetone and sonicated for 15 min to remove the residual photoresist. Finally, the notched silicon substrates were immersed in trimethoxy(octadecyl)silane solution (0.125% in hexane, v/v) for 20 min to form a silane passivation layer on the surface of the silicon substrate.

### Preparation of the templates with submicron notches

Commercial monodisperse PS spheres were mixed with different volume ratios of ethanol (C_2_H_6_O) and deionized water, depending on the diameter of the PS spheres. For example, the volume ratio of PS spheres with a nominal diameter of 1 μm was 1:2:1 (PS/H_2_O/C_2_H_6_O). The mixture was then sonicated and stirred for 10 min. Then the ordered PS monolayer was prepared by a micro-propulsion injection method, where about 0.5 mL of PS sphere solution was dropped to form an ordered PS monolayer at the air–water interface with an area of about 30 cm^2^. The PS sphere arrays were self-assembled on the silicon substrate with the gradual evaporation of the solvent as illustrated in Supplementary Fig. S12. The PS sphere arrays were then etched by ICP reactive ion etching (ICP-RIE, SI 500 Sentech) using a mixture of O_2_ and Ar gases. After the etching process, a gold thin film (15 nm) was deposited on the template by spraying equipment (Ei-5z, ULVAC). The PS spheres were then removed by sonication in tetrahydrofuran and chloroform for 10 min to obtain submicron patterned films. Then the fabricated submicron notches were immersed in trimethoxy(octadecyl)silane solution (0.125% in hexane, v/v) for 20 min to form a silane passivation layer on the substrate surface before use.

### Preparation of the QD patterns

For the preparation of aligned patterns, the QD solution containing a proper amount of DMS was first dropped on the patterned substrates equipped with two copper electrodes. The QD deposition process was illustrated in Supplementary Fig. S19. The AC electric field was generated by a high-voltage amplifier and oscilloscope (PINTECH, Guangzhou Deccan Electronics Co., Ltd.) connected to an HP function generator. The QD deposition was modulated by controlling the electric field intensity (0.8–8 V mm^−1^) and frequency (10 kHz–100 MHz) of the applied electric field. After that, the substrates were heated at 70 °C for 10 min and then washed with *n*-hexane to complete the fabrication process. The procedure for the preparation of QD pixels is similar to that of the aligned patterns. Differently, the orthogonal electric field was applied in the QD pixel fabrication process, where the electrodes were first applied to the electric field along the *X*-axis for 5 min and then applied to *Y*-axis electric field for another 5 min. For the QD pixel preparation in electroluminescent devices, the optimal DED parameters of QD concentration, deposition time, electric field frequency, and intensity are 25 mg mL^−1^, 10 min, 100 kHz, and 4 V mm^−1^, respectively.

### Fabrication of QD-patterned devices

The ITO glass substrates were successively cleaned by deionized water, acetone, and isopropyl alcohol, and then treated with a UV-ozone apparatus for 20 min. Then ZnMgO solution (25 mg mL^−1^ in ethanol) was spin-coated on the substrates at 2000 rpm for 60 s and annealed at 120 °C for 30 min. After that, poly(methylmethacrylate) (PMMA) solution (5 mg mL^−1^ in acetone) was spin-coated on the substrates at 4000 rpm for 60 s and annealed at 80 °C for 10 min. Then DL-isoborneol solution (5 mg mL^−1^ in ethanol) was spin-coated at 2000 rpm for 60 s, followed by the preparation process of photoresist patterns. Then the PMMA layer without the coverage of the photoresist was completely etched and removed using an ICP-RIE (SI 500 Sentech) equipment followed by the removal process of the photoresist. After the preparation of PMMA notch pattern, the QDs (25 mg mL^−1^ in *n*-hexane) were deposited on the patterned substrate by the orthogonal electric field as shown above, followed by a mild heating process at 70 °C for 10 min to form PDMS layer between QD patterns. After that, a layer of TFB (8 mg mL^−1^ in chlorobenzene) was spin-coated at 3000 rpm for 40 s and annealed at 120 °C for 15 min. It is noted that the chlorobenzene solvent can damage the PMMA and QD layers, resulting in the direct contact between ZnMgO ETL and TFB HTL for large leakage current. While the PDMS layer avoids the erosion of chlorobenzene, serving as a charge barrier layer for good suppression of the leakage current. Subsequently, MoO_3_ (8 nm) and Al (100 nm) were deposited at the deposition rate of 0.4 and 1 Å s^−1^ by thermal evaporation under a vacuum of 5 × 10^−4^ Pa to complete the device fabrication process.

### QD pattern and device characterization

UV–vis absorption spectra were recorded on an Agilent Technologies 8453 UV–vis spectrophotometer. FT-IR spectra were obtained on a Tensor 27 FT-IR spectrophotometer. PL emission spectra of the QDs were measured on an Edinburgh FS5 fluorescence spectrometer. PLQY was measured on an Edinburgh FS5 with an integrating sphere. Transmission electron microscopy micrographs were taken on an F20 (FEI Tecnai, Netherlands). XRD patterns were recorded on HR-XRD (D8 discover, Bruker). Dynamic SIMS data were collected by TOF.SIMS-5 (ION-TOF, Germany). Exposure of the photoresist was carried out under photolithography equipment (ABM/6/350/NUV/DCCD/BSV/M, USA). The performance of the devices was measured by the ocean optical system, which consists of a power meter (Keithley 2450), a spectrometer (QE Pro), and an integrating sphere (FOIS-1).

## Supplementary information

supplemental information

## Data Availability

The data that support the findings of this study are available from the corresponding author upon reasonable request.
