# Supplementary material for: Ultrahigh-resolution, high-fidelity quantum dot pixels patterned by dielectric electrophoretic deposition
Source: Light Sci Appl. 2024 Sep 26;13:273. doi: 10.1038/s41377-024-01601-3 (PMC11427692; doi:10.1038/s41377-024-01601-3)
Supplement: Supplementary file 1 — supplemental information [file 41377_2024_1601_MOESM1_ESM.docx]

**Supplementary Information for**

**Ultrahigh-resolution, high-fidelity quantum dot pixels patterned by dielectric electrophoretic deposition**

Chengzhao Luo^1^, Yanhui Ding^1^, Zhenwei Ren^1,^*, Chenglong Wu^1^, Yonghuan Huo^1^, Xin Zhou^1^, Zhiyong Zheng^1^, Xinwen Wang^1^, Yu Chen^1,2,^*

^1^School of Optoelectronic Science and Engineering & Collaborative Innovation Center of Suzhou Nano Science and Technology, Soochow University, Suzhou 215006, China E-mail: [zhwren@suda.edu.cn](mailto:zhwren@suda.edu.cn); [chenyu_ny@suda.edu.cn](mailto:chenyu_ny@suda.edu.cn)

^2^National University of Singapore Suzhou Research Institute, Dushu Lake Science and Education Innovation District, Suzhou 215123, P. R. China

Capacitance-Frequency (*C*-*F*) measurement.


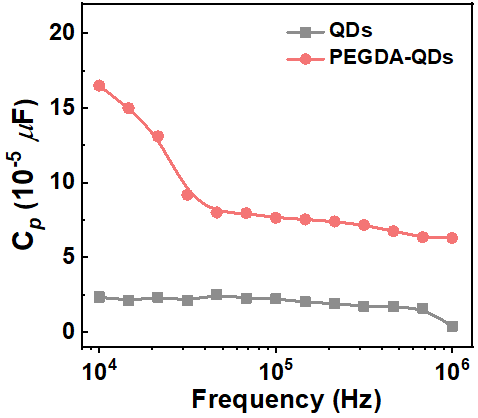
The dielectric constant of QDs and PEGDA decorated QDs can be extracted from the *C*-*F* measurement with the equation of *ε*= *C d*/(*ε*_0_ *S*), in which *ε*_0_ is the vacuum permittivity, *S* is the device area, and *d* is the thickness of the perovskite film. In the equation, the higher value of *C* indicates the larger ε when d and S are the constant. From the *C*–*F* curves, the low values of capacitance for QDs suggest the small dielectric constant for QDs. While the capacitance is greatly improved for PEGDA-QDs, indicating an enlarged dielectric constant for PEGDA-QDs.

**Supplementary Fig. S1** The *C*–*F* plot of the devices with the structures of ITO/QDs/Al and ITO/PEGDA-QDs/Al. The measurement was performed with an AC amplitude of 25 mV.

**
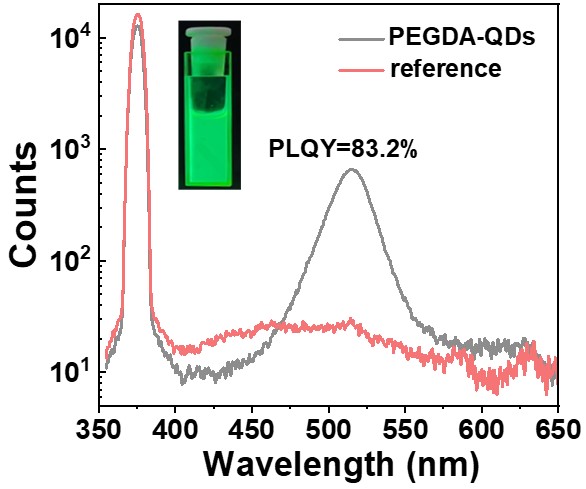
**

**Supplementary Fig. S2** Photoluminescence quantum yield (PLQY) measurement of PEGDA-QDs (inset: the picture of QDs solution under UV light).

**
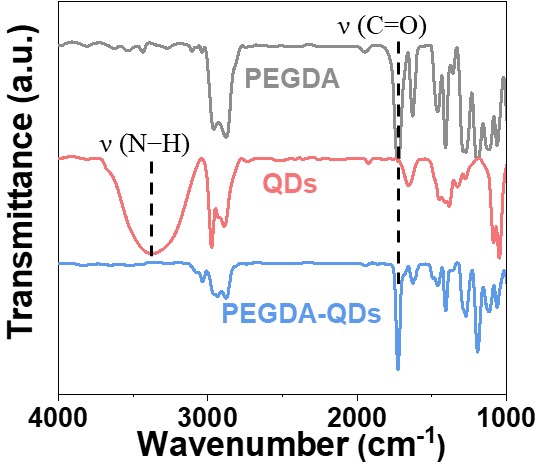
**

**Supplementary Fig. S3** FT-IR of PEGDA, QDs, and PEGDA decorated QDs.


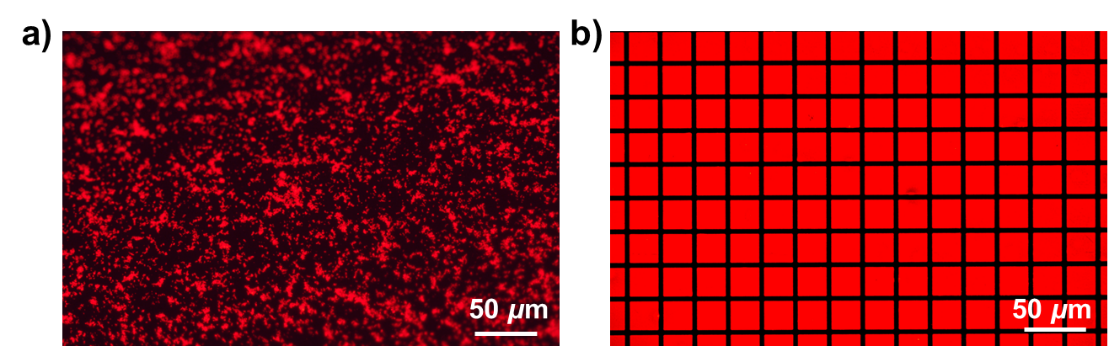


**Supplementary Fig. S4** Fluorescence microscopy images of a) pristine CdSe QDs and b) PEGDA decorated QDs after dielectric electrophoretic deposition.


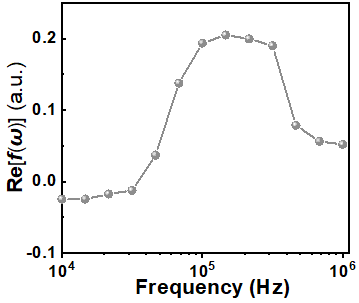


**Supplementary Fig. S5** Plot of Re[f(ω)] *versus* the electric field frequency.


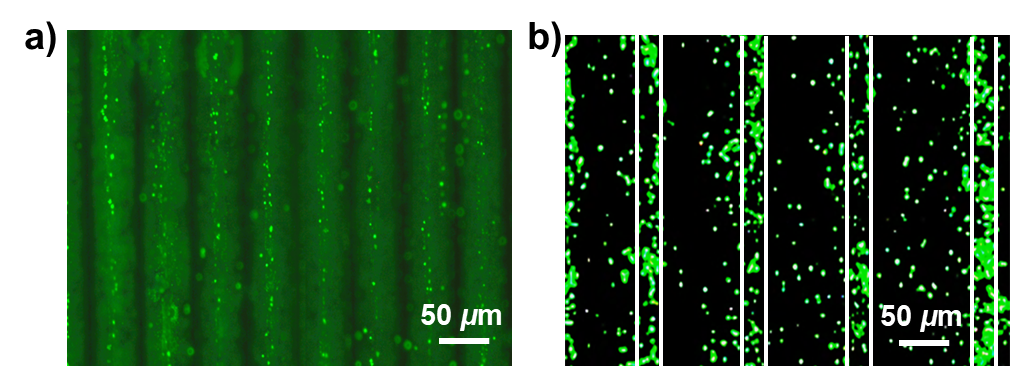


**Supplementary Fig. S6** Fluorescence microscopy images of QD aligned patterns driven under the electric field intensity of a) 4 V mm^-1^ and b) 8 V mm^-1^, respectively.


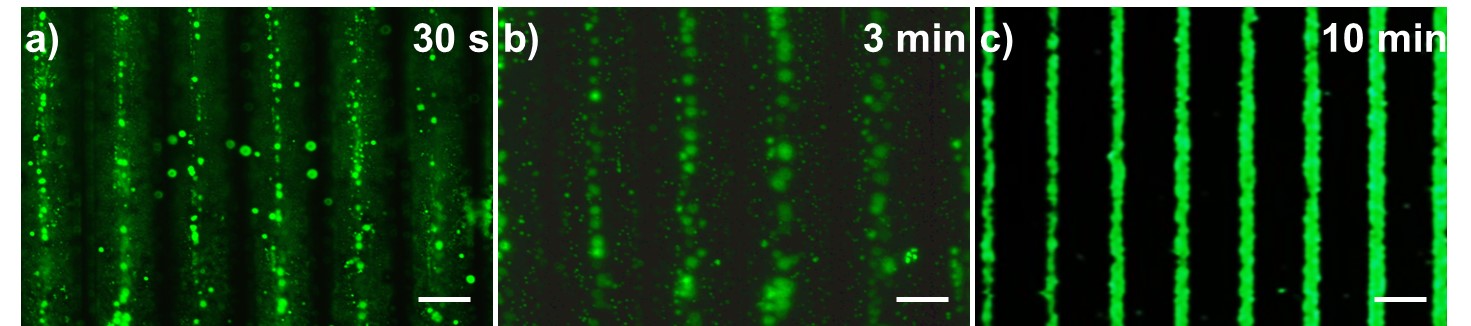


**Supplementary Fig. S7** Fluorescence microscopy images of QD patterns at different deposition times: a) 30 s, b) 3 min, and c) 10 min. The scale bar is 50 *μ*m in each picture.


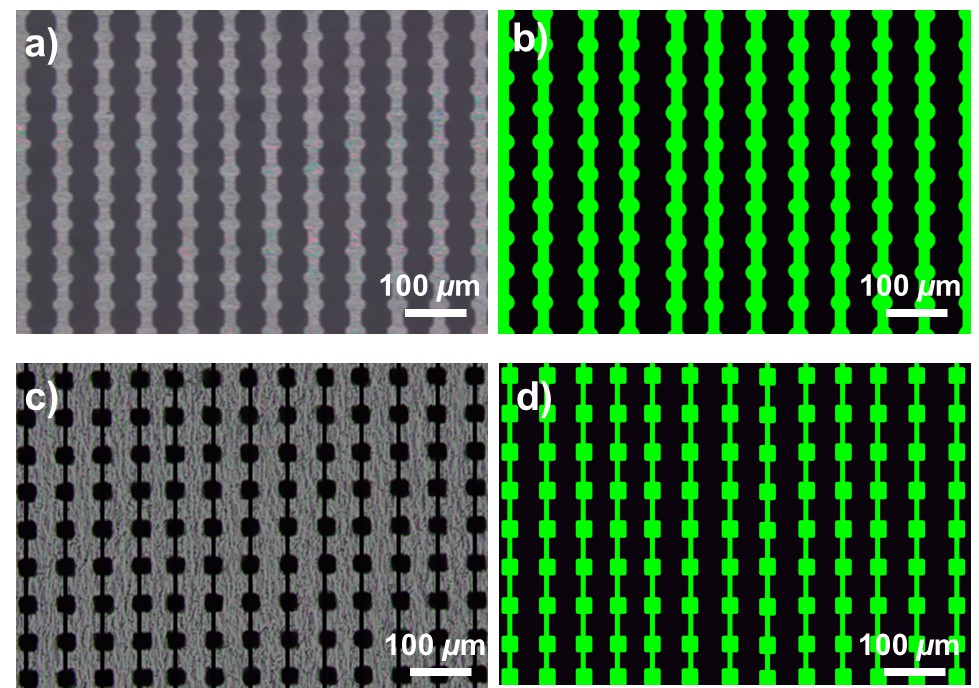


**Supplementary Fig. S8** The pictures of the striped templates with a) circle and c) square geometric shapes, and the fluorescence microscopy images of QD stripe patterns based on b) circle and d) square striped templates fabricated by template-assisted DED method.


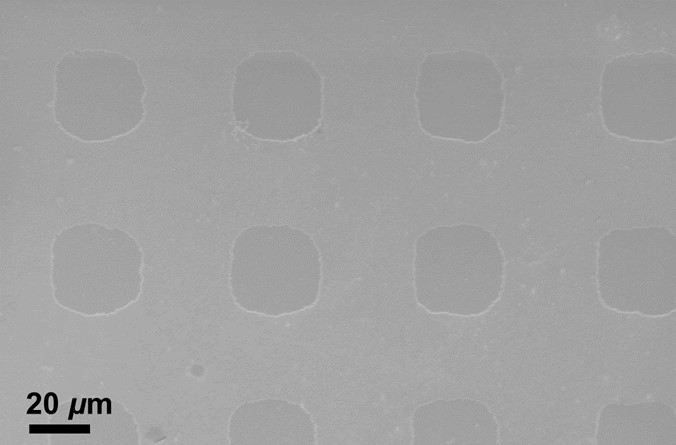


**Supplementary Fig. S9** Microscopy image of square-shaped templates.

**
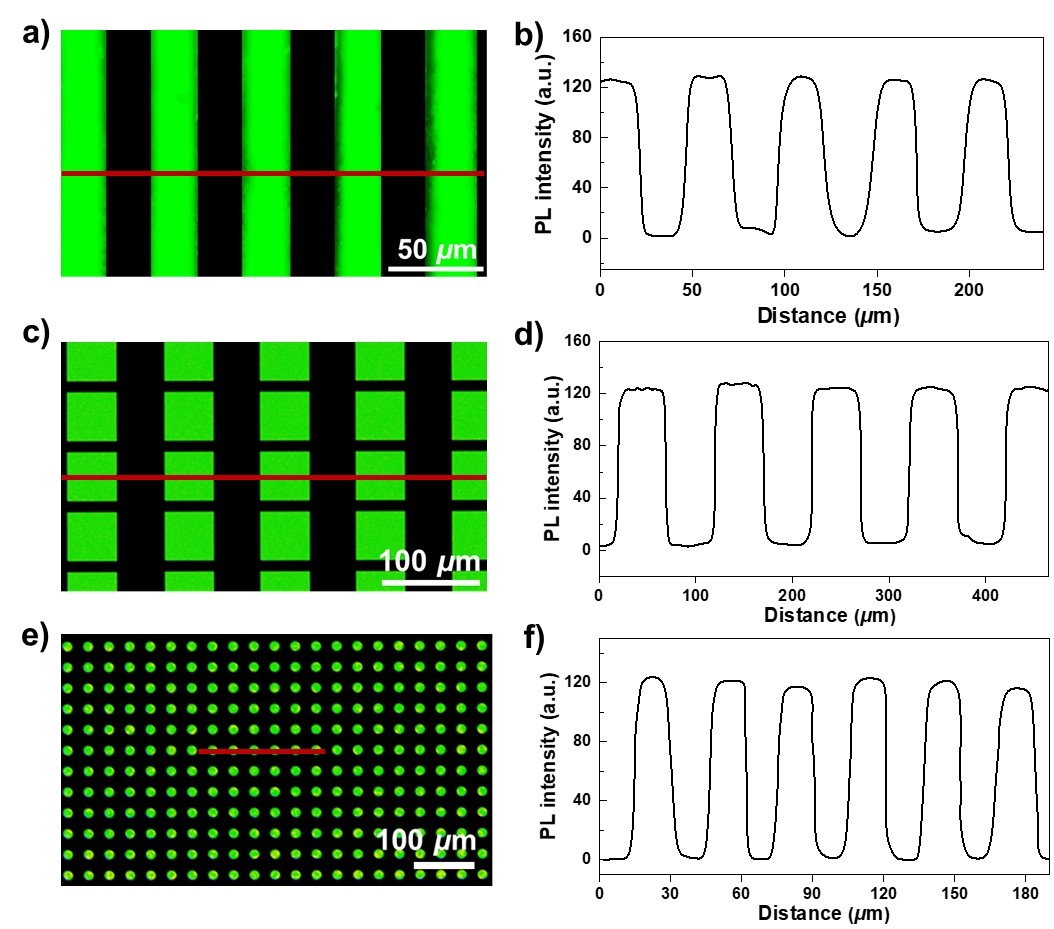
**

**Supplementary Fig. S10**. Fluorescence microscopy images of a) QD striped patterns, c) square pixels (289 PPI), and e) circle pixels (1104 PPI), and b, d, f) their corresponding PL intensities along the red lines from the detected region.


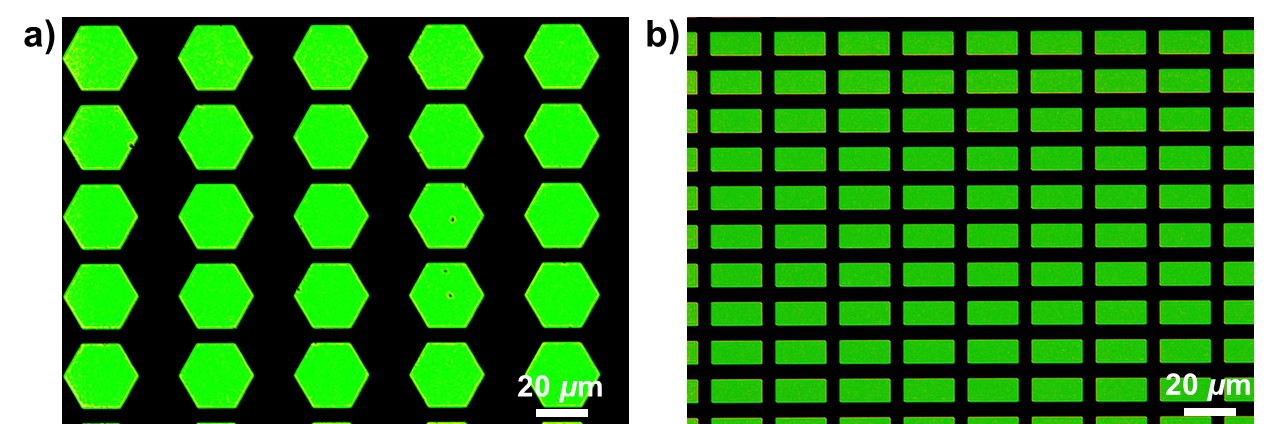


**Supplementary Fig. S11** Fluorescence microscopy images of QD pixels with a) regular hexagons and b) rectangle shapes.


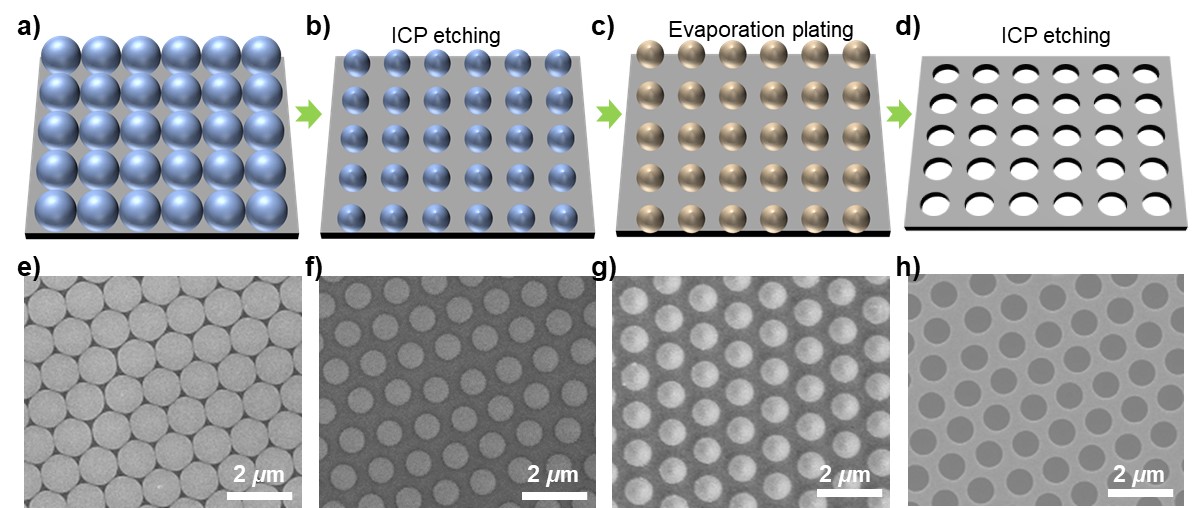


**Supplementary Fig. S12** Illustration of the fabrication process of submicron notches with the polystyrene (PS) sphere-masked technique: a, e) polystyrene (PS) sphere arrangement, b, f) inductively coupled plasma (ICP) etching, c, g) evaporation plating, and d, h) ICP etching for the submicron notches.


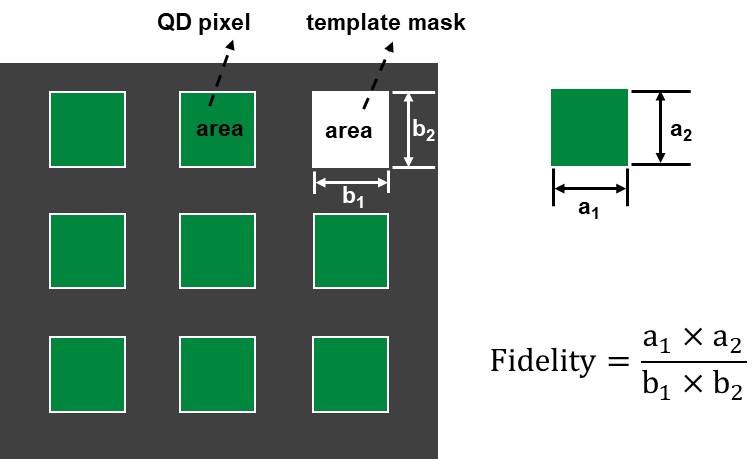


**Supplementary Fig. S13**. Illustration of the QD pixel fidelity calculation (e.g., the rectangular QD pixel). The green and white areas represent a single QD pixel and corresponding void in template mask, respectively. The fidelity is defined as the ratio of the green area to the white area.


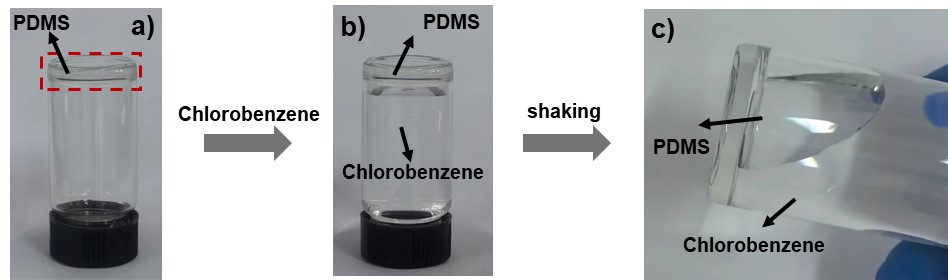


**Supplementary Fig. S14** The pictures of a) PDMS gel (in the dotted box), b) PDMS gel and chlorobenzene solution, and c) the PDMS and chlorobenzene solution after being immersed into chlorobenzene for several hours.


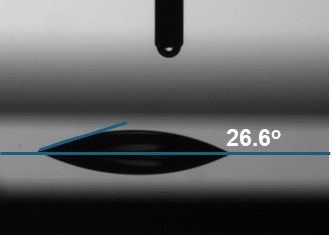


**Supplementary Fig. S15** The contact angle measurement of chlorobenzene on PDMS film.


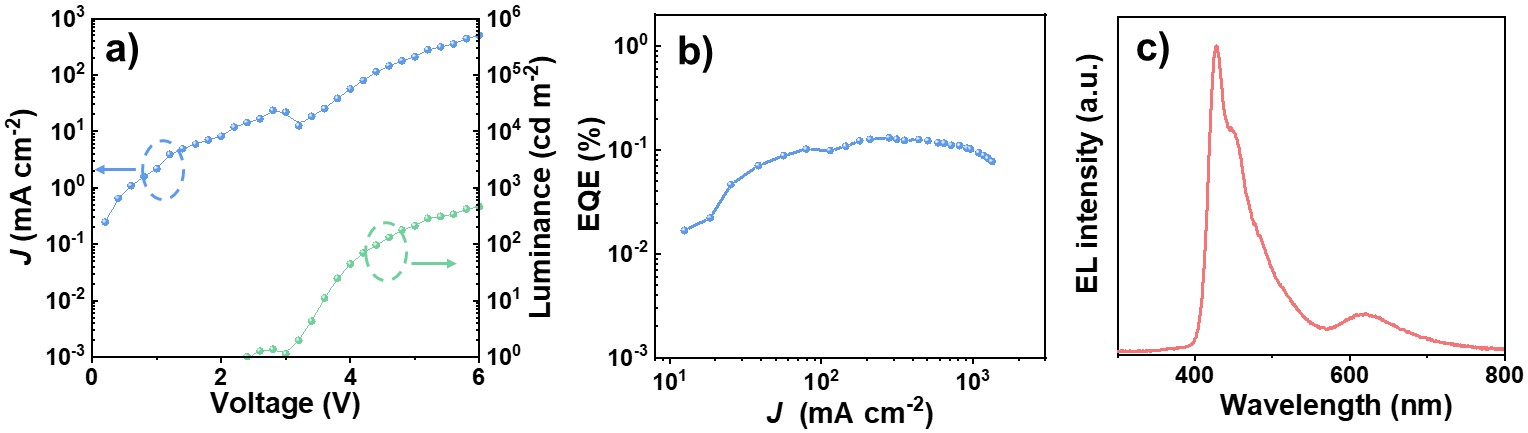


**Supplementary Fig. S16** The performances of device without PDMS: a) current density-luminance-voltage (*J*-*L*-*V*) and b) efficiency-current density (*EQE*-*J*) curves, and c) the corresponding EL spectrum.


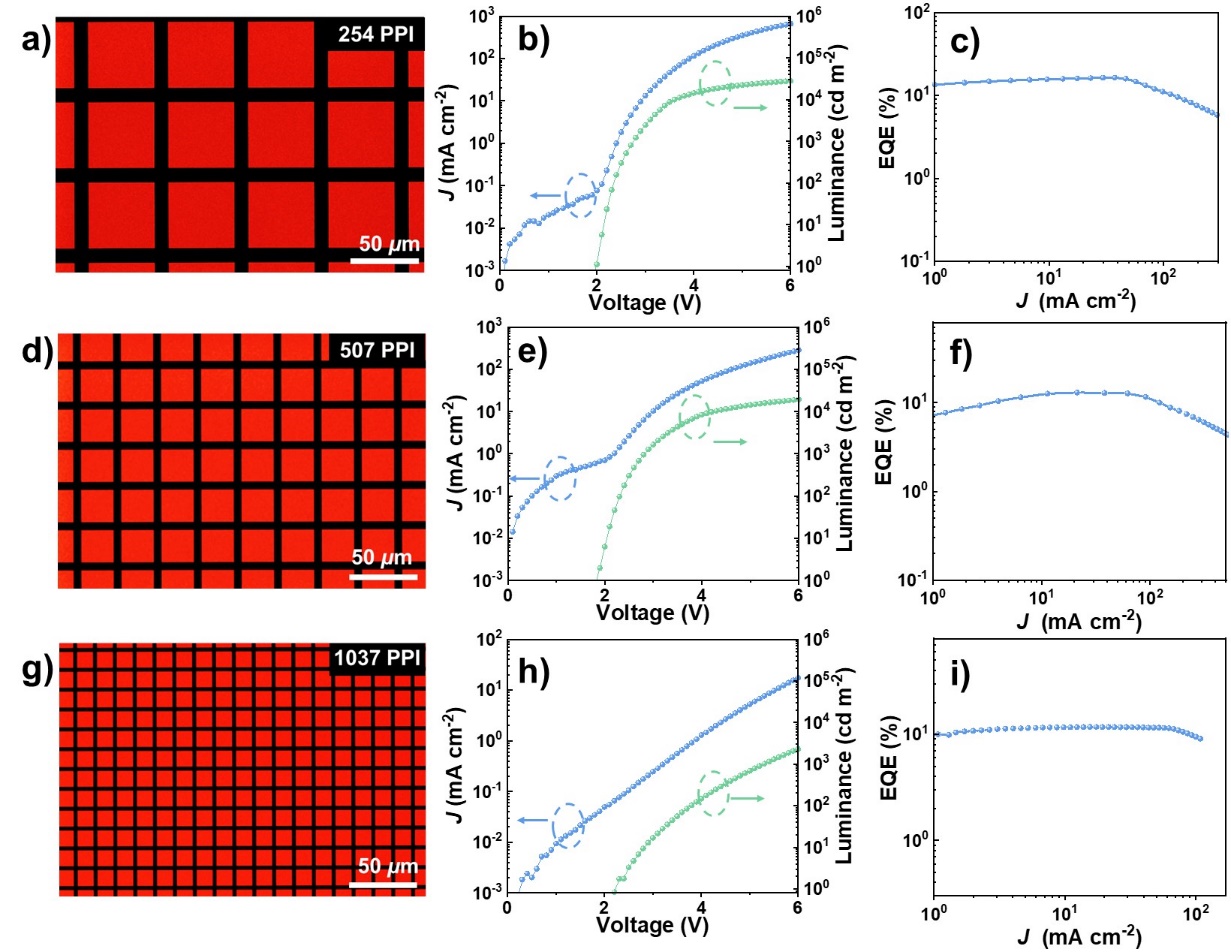


**Supplementary Fig. S17** Fluorescence microscopy images of red CdSe QD pixels with the resolutions of a) 254, d) 507, and g) 1037 PPI, and their corresponding device performances: b, e, h) current density-luminance-voltage (*J*-*L*-*V*) and c, f, i) efficiency-current density (*EQE*-*J*) curves. The dimethylsiloxane oligomer concentrations of 0.5, 1.2, and 2.2 mg mL^-1^ were adopted to prepare the QD pixels with the resolutions of 254, 507, and 1037 PPI, respectively, to suppress the current leakage.


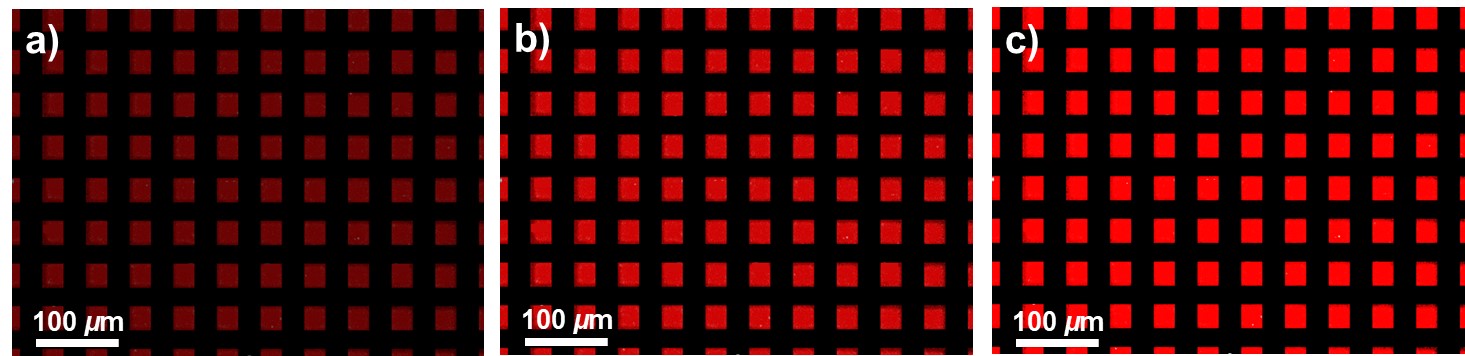


**Supplementary Fig. S18** Optical microscopy images of CdSe QD pixel device under the applied voltages of a) 2.1, b) 2.2, and c) 2.4 V. The QD pixel image at higher voltages is too bright to be detected.


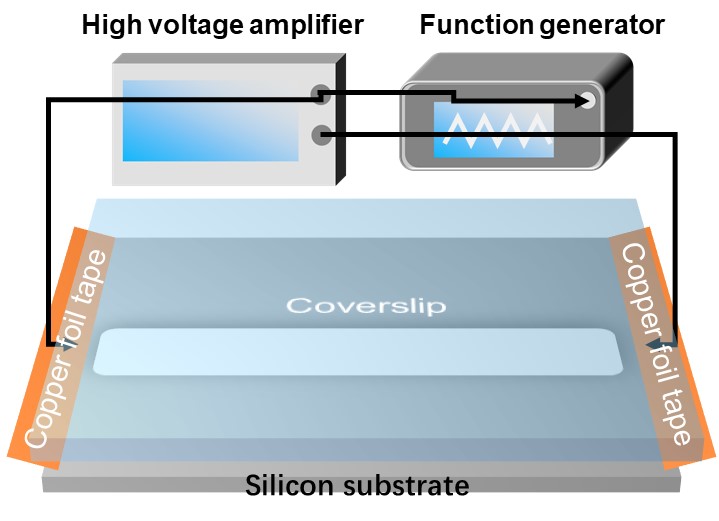


**Supplementary Fig. S19** Illustration of the dielectric electrophoretic deposition of QD patterns.

**Supplementary Table**

**Supplementary Table S1.** The Zeta potential measurements for CdSe and CsPbBr_3_ QDs.

| QDs | Zeta potential (mV) |
| --- | --- |
| CdSe QDs | -28 |
| CsPbBr_3_ QDs | -18 |
